# Potential mechanism for hyperhomocysteinemia in Greyhound dogs

**DOI:** 10.1111/jvim.16700

**Published:** 2023-04-24

**Authors:** Kelsey L. Johnson, Torrey Tiedeman, Hannah Peterson, Joerg M. Steiner, Lauren A. Trepanier

**Affiliations:** ^1^ Department of Medical Sciences University of Wisconsin‐Madison Madison Wisconsin USA; ^2^ Gastrointestinal Laboratory, Department of Small Animal Clinical Sciences Texas A&M University College Station Texas USA

**Keywords:** cobalamin, folate, methionine synthase, oxidative stress, sighthounds

## Abstract

**Background:**

Greyhounds have been reported to have hyperhomocysteinemia (HHC), but the underlying mechanisms and clinical implications are unclear.

**Hypothesis:**

Our primary aim was to assess serum concentrations of homocysteine (HCy) and related analytes in Greyhounds and to identify a likely metabolic pathway for HHC. A secondary aim was to determine whether HHC is associated with evidence of oxidative stress.

**Animals:**

Healthy pet Greyhounds (n = 31) and non‐sighthound control dogs (n = 15).

**Methods:**

Analysis of serum HCy, cobalamin, folate, and methionine, and plasma cysteine, glutathione, and total 8‐isoprostane concentrations.

**Results:**

Homocysteine concentrations were higher in Greyhounds (median, 25.0 μmol/L) compared to controls (13.9 μmol/L; *P* < .0001). Cobalamin concentrations were lower in Greyhounds (median, 416 ng/L) compared to controls (644 ng/L; *P* = .004) and were inversely correlated with HCy (*r* = −0.40, *P* = .004). Serum concentrations of folate, which is regenerated when HCy is converted to methionine, also were inversely correlated with HCy (*r* = −0.47, *P* = .002). Serum methionine concentrations were more than 4‐fold lower in Greyhounds (median, 3.2 μmol/L) compared to controls (median, 15.0 μmol/L), but this difference was not significant (*P* = .3). Plasma cysteine, glutathione, and 8‐isoprostane concentrations did not differ significantly between groups.

**Conclusions and Clinical Importance:**

Our findings suggest a primary defect in conversion of HCy to methionine in Greyhounds, with related impaired folate generation. Ineffective cycling by methionine synthase could lead to secondary cobalamin depletion. Notably, low serum folate and cobalamin concentrations can be observed in Greyhounds without signs of intestinal disease.

**Abbreviations**
HHC: hyperhomocysteinemia
HCy: homocysteine

## 1 INTRODUCTION

Homocysteine (HCy) is a nonessential amino acid and precursor of both methionine and cysteine. Hyperhomocysteinemia (HHC) in people is strongly associated with some chronic disorders such as cardiovascular disease, stroke, macular degeneration, and cognitive impairment.^1^ Hyperhomocysteinemia leads to oxidative stress experimentally in rodents and dogs.^2^, ^3^, ^4^ Homocysteine can be oxidized at its free thiol, which can lead to downstream protein, DNA, and lipid oxidation.^5^ In human patients, serum HCy concentrations correlate with oxidative stress, as measured by increases in plasma isoprostane concentrations.^6^, ^7^, ^8^, ^9^ Oxidative stress is a mechanism by which HHC leads to disease pathogenesis in human patients.^5^

**FIGURE 1:**
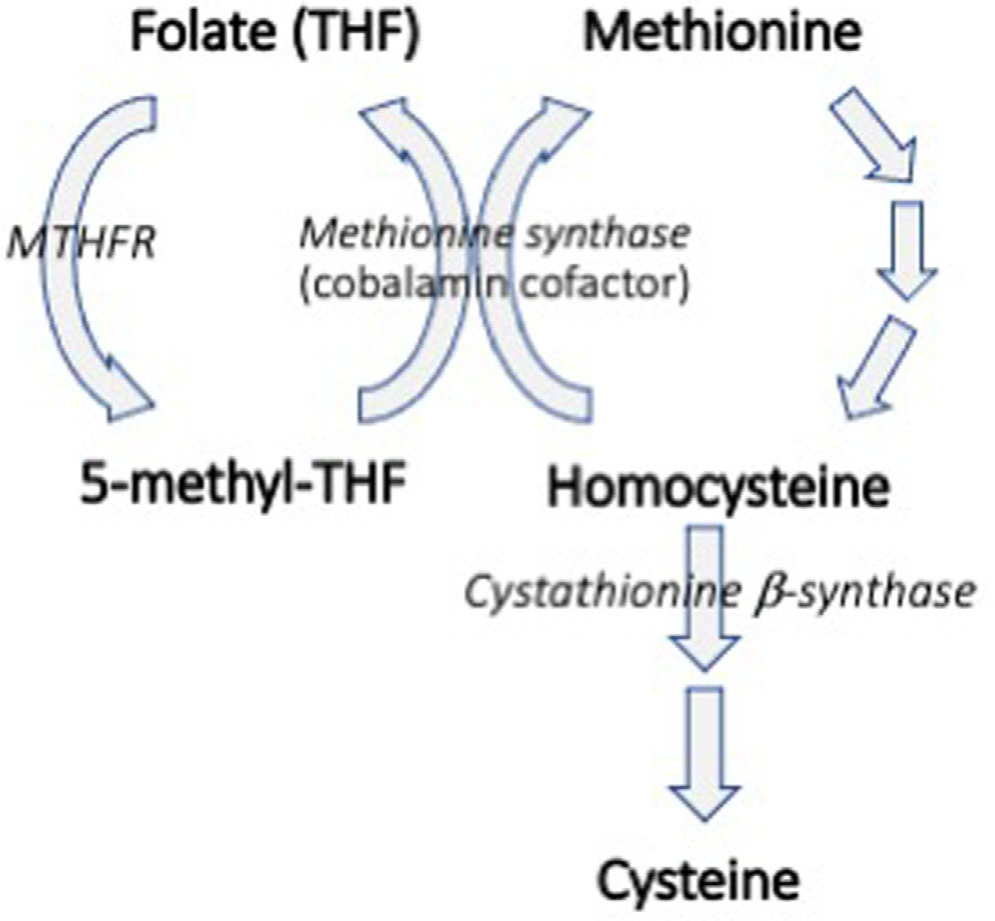
Homocysteine is an amino acid intermediate in the synthesis of methionine. Homocysteine is also an indirect precursor of the amino acid cysteine. In people, hyperhomocysteinemia results from deficiencies in B vitamins such as folate or cobalamin, or from genetic defects in the enzymes methylenetetrahydrofolate reductase (MTHFR), methionine synthase, or cystathionine β‐synthase.^1^ (Figure adapted from references 10 and 14).

In people, HHC can result from genetic defects in the enzymes catalyzing HCy interconversion or from acquired deficiencies in folate (vitamin B9) or cobalamin (vitamin B12),^1^ which are necessary to drive conversion of HCy to methionine (Figure 1). Whether because of a heritable or acquired disorder, HHC can respond to B vitamin supplementation in human patients.^10^, ^11^, ^12^, ^13^ In dogs, HHC has been reported in cobalamin‐deficient Shar Pei dogs, but response to cobalamin supplementation has not been evaluated.^14^

Greyhounds have an apparent breed‐related syndrome of HHC.^15^ In a prior study in 16 clinically healthy Greyhounds, serum HCy concentrations were all above‐reported reference intervals (>22.1 μmol/L),^14^ with approximately 4‐fold individual variability.^15^ Notably, serum HCy concentrations were all in a range associated with increased risk for adverse outcomes in humans (>15 μmol/L), particularly increased risk for cardiovascular disease or stroke.^5^ Given that Greyhounds are reportedly at higher risk for ischemic strokes compared to other dog breeds,^16^ it is important to understand the mechanisms and clinical implications of HHC in Greyhounds.

Our primary aim was to assess serum concentrations of HCy, folate, cobalamin, methionine, and cysteine in healthy Greyhound dogs to identify likely metabolic pathways associated with HHC. A secondary aim was to determine whether HHC in clinically healthy Greyhounds is associated with evidence of oxidative stress, as measured by total plasma concentrations of 8‐isoprostanes.

## 2 MATERIALS AND METHODS

### 2.1 Dog recruitment and eligibility

Clinically healthy pet Greyhounds, mostly retired racing dogs, were screened for inclusion in the study. Owners provided written informed consent before screening. Clinical histories, including current diet, medications, and supplements, were obtained from all dogs. Dogs were eligible if they were at least 1 year of age, were eating a balanced commercial dog food, and had no evidence of systemic illness on history, physical examination, CBC, or serum biochemical panel. Dogs were ineligible if they were eating a raw diet or grain‐free food, had undergone a diet change in the past month, were receiving antioxidants or dietary supplements of any kind, or were on any medications other than monthly heartworm and flea preventatives.

Clinically healthy non‐Greyhound, non‐sighthound dogs, at least 1 year of age and of comparable sex, neuter status, and age (±1 year) as the Greyhounds, also were screened for inclusion in the study, using the same criteria used for the Greyhounds. All dogs were cared for under guidelines approved by the University of Wisconsin‐Madison Institutional Animal Care and Use Committee.

### 2.2 Study interventions

At the time of screening, an additional 16 mL of blood was collected (10 mL divided into 2 5‐mL additive‐free serum tubes and 6 mL divided into 3 2‐mL heparinized tubes) for testing associated with the study. Dogs were fasted overnight before blood collection. Serum was separated and frozen at −20°C for analysis of HCy, cobalamin, folate, and methionine. Heparinized blood was placed on ice and immediately treated by addition of bromobimane to 2 tubes (200 μL of 27 mM stock solution per 2 mL blood) to stabilize plasma cysteine and glutathione,^17^ and BHT to the third tube (20 μL of 0.5% stock solution per 2 mL) to stabilize plasma isoprostanes.^18^ Plasma was harvested and frozen at −80°C until analyses for cysteine, glutathione, and total 8‐isoprostane concentrations.

Serum folate and cobalamin concentrations were measured using analytically validated chemiluminescent assays (Immulite 2000 platform).^19^, ^20^ Serum HCy and methionine concentrations were measured using gas chromatography/mass spectrometry (GC/MS) assays, also analytically validated for use in dogs.^11^, ^21^ These assays all were performed at the Gastrointestinal Laboratory at Texas A&M University. Plasma cysteine was assayed by high‐performance liquid chromatography (HPLC) with bromobimane tagging and fluorescence detection as previously reported by our laboratory.^22^ Total plasma 8‐isoprostane concentrations were measured using a standard competitive enzyme immunoassay kit (Cayman Chemical Company, Ann Arbor, Michigan)^18^, ^23^ on a fee‐for‐service basis through Cayman Chemical.

### 2.3 Statistical analyses and sample size

Data were assessed for normality using Shapiro‐Wilk tests. Serum HCy, methionine, folate, and cobalamin and plasma cysteine, glutathione, and 8‐isoprostane concentrations were compared between healthy pet Greyhounds and non‐sighthounds using Mann‐Whitney *U* tests. Comparisons of proportions were performed using Fisher's exact tests. Serum HCy concentrations were correlated with age, plasma 8‐isoprostanes, and other biomarkers using Spearman rank correlation. All statistical analyses were performed using commercially available software (Prism 9, GraphPad Software, San Diego, California).

Based on significant increases in plasma F2‐isoprostane concentrations observed in humans with HHC compared to controls,^7^ we estimated that a sample size of 30 Greyhounds and 15 controls would provide 90% power to detect similar increases in plasma F2‐isoprostane concentrations in Greyhounds, with *P* < .05. As few as 34 dogs total (Greyhound and controls) also would provide adequate power to detect a significant positive correlation of at least 0.47 between HCy and F2‐isoprostane concentrations as previously observed in human patients.^8^ Sample size calculations were performed using an open‐source calculator (http://biomath.info).

## 3 RESULTS

### 3.1 Dog demographics

The median age of both pet Greyhound dogs (n = 31) and non‐sighthound controls (n = 15) was 3.5 years, and sex and neuter status were comparable between groups (Table 1). An additional 5 dogs (3 Greyhounds and 2 controls) were screened for the study but were ineligible because of abnormalities in medical history or screening blood test results (e.g., mild azotemia, eosinophilia, history of chronic enteropathy).

**TABLE 1:** Demographic data for healthy pet dogs enrolled in a prospective observational study of serum homocysteine concentrations and related biomarkers.

	Greyhounds (n = 31)	Non‐sighthound controls (n = 15)
Median age (range)	3.5 years (1.5‐12.5)	3.5 years (1.5‐7.5)
Sex	Female spayed 12 Male neutered 19	Female spayed 5
		Female intact 1
		Male neutered 9
Breed	All Greyhounds	Mixed breed (5)
		Golden retriever (3)
		Australian shepherd (2)
		Labrador retriever (1)
		Border collie (1)
		American pit bull terrier (1)
		German shorthaired pointer (1)
		Treeing Walker coonhound (1)

### 3.2 Homocysteine, cysteine, and glutathione concentrations

**FIGURE 2:**
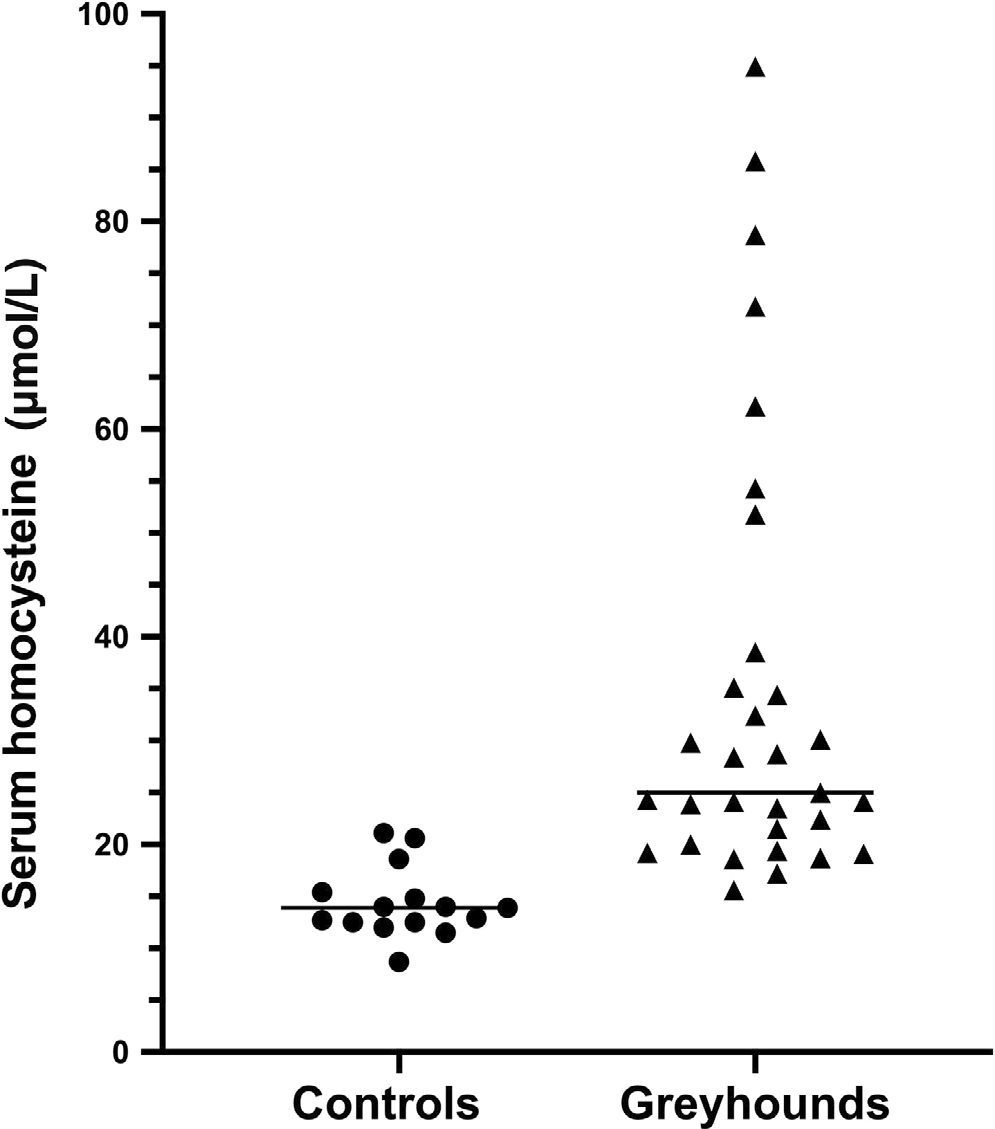
Serum homocysteine concentrations in pet Greyhound dogs and non‐sighthound controls. Homocysteine concentrations were significantly higher in Greyhound dogs (*P* < .0001). In this and subsequent scatter plots, each point represents 1 dog, and horizontal bars indicate the group medians.

As expected, serum HCy concentrations were significantly higher in Greyhound dogs (median, 25.0 μmol/L; range, 15.6‐94.9 μmol/L) compared to non‐sighthound controls (median, 13.9 μmol/L; range, 8.7‐21.2 μmol/L; *P* < .0001; Figure 2). For context, reported reference intervals for serum HCy concentrations in healthy pet dogs (breeds not specified) were 4.3‐18.4 μmol/L^24^ and 5.0‐22.1 μmol/L.^14^ In our study, serum HCy concentrations were positively correlated with age across Greyhounds (*r* = 0.42; 95% confidence interval [CI] 0.07‐0.68; *P* = .02). This same relationship was found in the smaller non‐Greyhound control population, but did not reach significance (*r* = 0.42, 95% CI, −0.16 to 0.79; *P* = .13).

**FIGURE 3:**
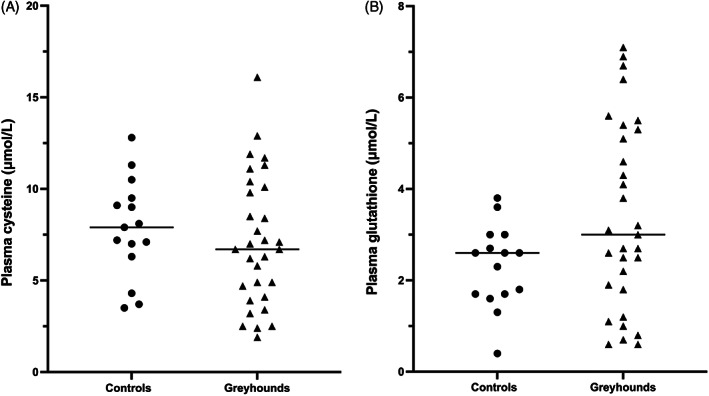
Plasma cysteine (A) and plasma glutathione (B) concentrations in pet Greyhound dogs and non‐sighthound controls. No significant differences were found between groups (*P* = .36 and .11, respectively).

Plasma cysteine and glutathione concentrations did not differ significantly between breed groups. Median plasma cysteine concentrations were 6.7 μmol/L in Greyhounds and 7.9 μmol/L in controls (Figure 3A; *P* = .36) and median plasma glutathione concentrations were 3.0 μmol/L in Greyhounds and 2.6 μmol/L in controls (Figure 3B; *P* = .11).

### 3.3 Serum cobalamin, folate, and methionine concentrations

**FIGURE 4:**
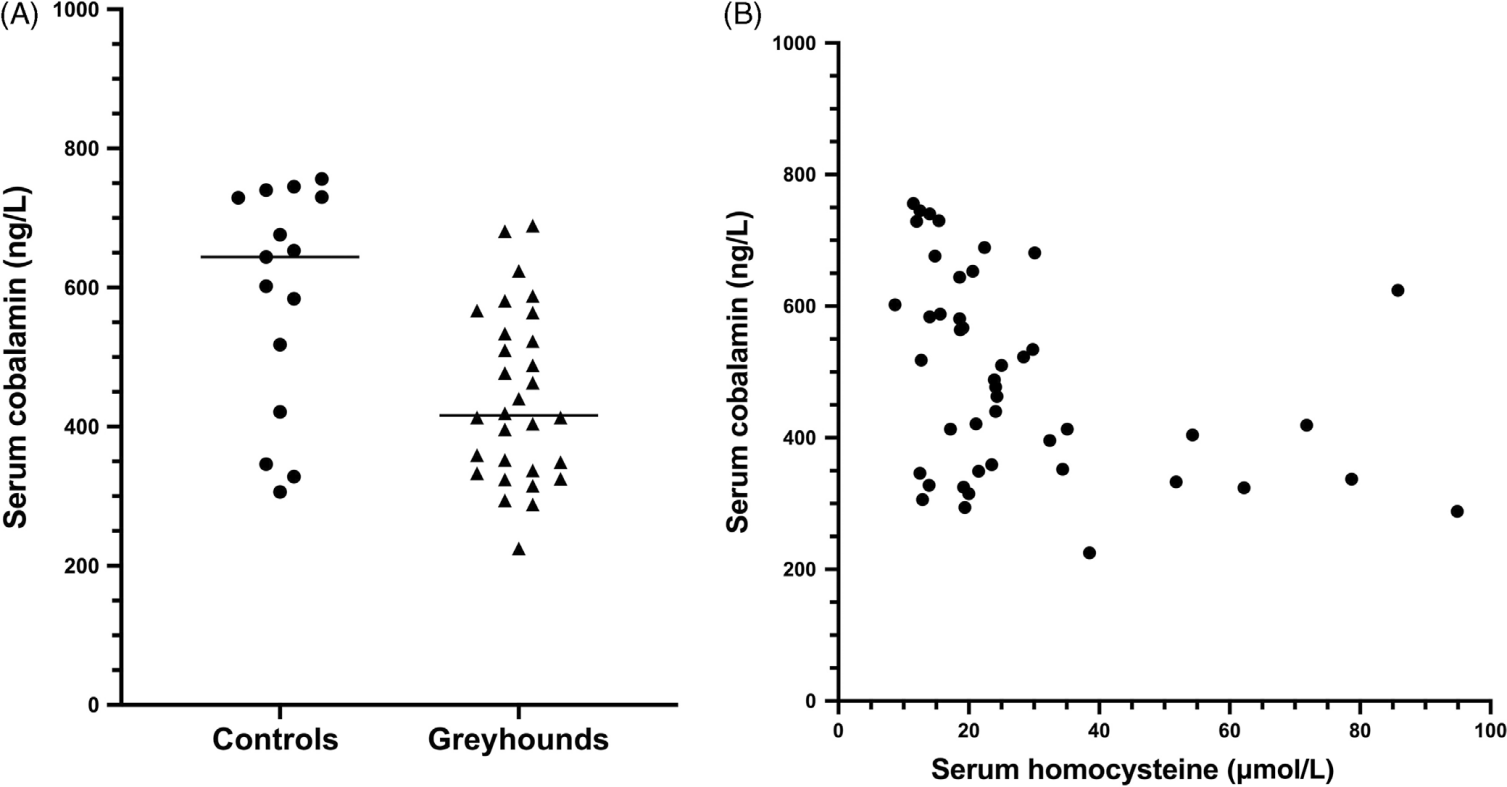
(A) Serum cobalamin concentrations in pet Greyhound dogs and non‐sighthound controls, which were significantly lower in Greyhounds (*P* = .004). (B) Significant inverse correlation between serum cobalamin and homocysteine concentrations across all dogs (*r* = −0.43, *P* = .004).

Concentrations of serum cobalamin, which is a necessary cofactor for conversion of HCy to methionine, were significantly lower in Greyhounds (median, 416 ng/L; range, 225‐689 ng/L) compared to control dogs (median, 644 ng/L; range, 306‐756 ng/L; *P* = .004; Figure 4A); the laboratory reference interval for serum cobalamin concentration in dogs is 251‐908 ng/L. In addition, serum cobalamin concentrations were significantly and inversely correlated with serum HCy concentrations across all dogs (*r* = −0.40, *P* = .004; Figure 4B). However, although 12 of the 31 Greyhounds (39%) had serum cobalamin concentrations in the low reference range at which supplementation is recommended (<400 ng/L), only 1 had cobalamin concentrations below the lower limit of the reference interval of 251 ng/L.

**FIGURE 5:**
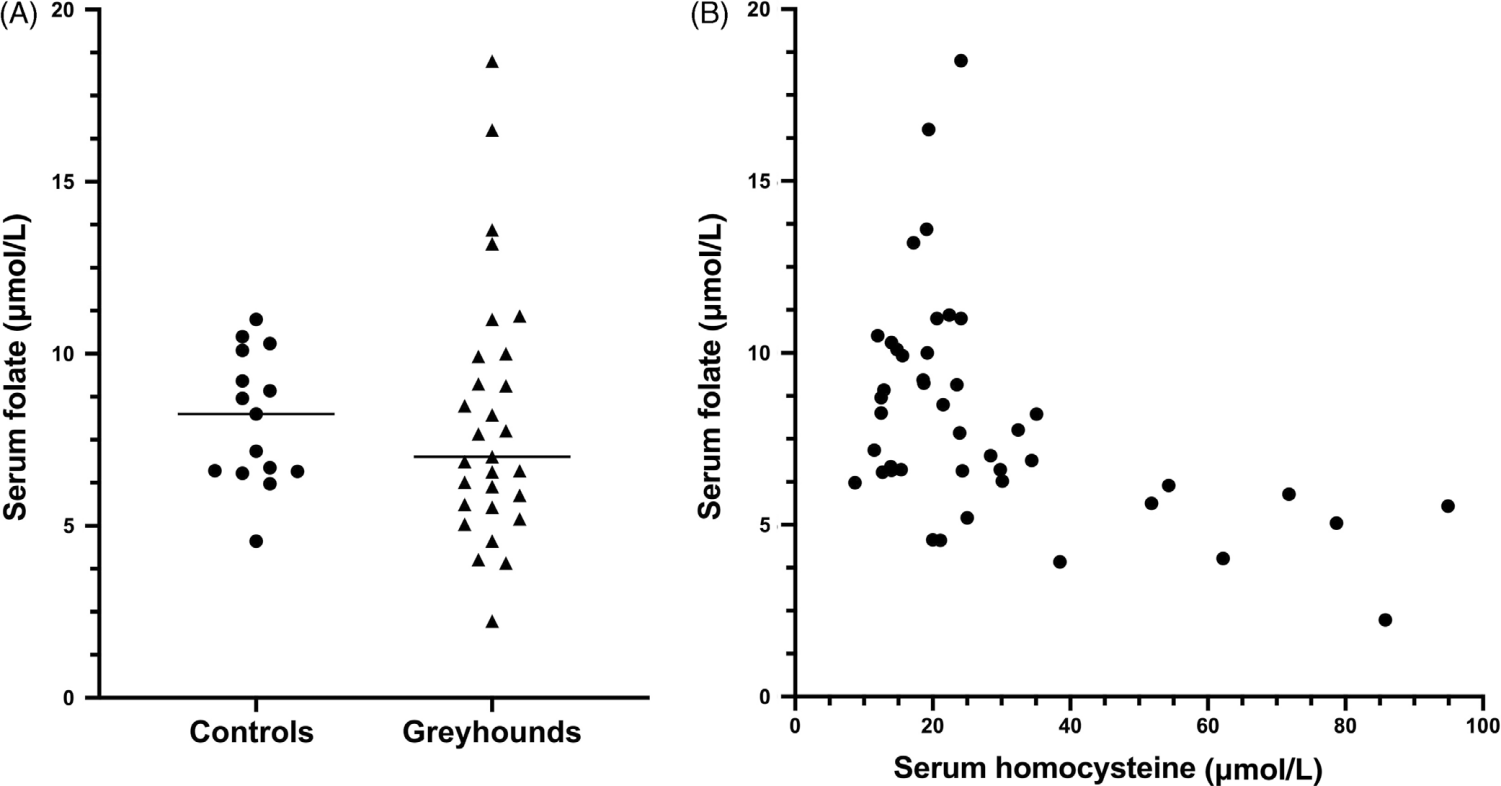
(A) Serum folate concentrations in pet Greyhound dogs and non‐sighthound controls, which were not significantly different between groups (*P* = .49). (B) However, a significant inverse correlation was found between serum folate and homocysteine concentrations across all dogs (*r* = −0.47, *P* = .002).

Concentrations of serum folate, which is regenerated when HCy is converted to methionine, were modestly but not significantly lower in Greyhounds (median, 7.0 μg/L) compared to controls (median, 8.3 μg/L; *P* = .49; Figure 5A). Two Greyhound samples had insufficient serum volume to measure folate. Overall, 55.2% of the Greyhounds (16 of 29) had serum folate concentrations below the laboratory reference interval of 7.7 to 24.4 μg/L, which was not significantly different from controls (46.7%; 7 of 15; *P* = .75). However, like cobalamin, serum folate concentrations were inversely correlated with serum HCy concentrations across all dogs (*r* = −0.47, *P* = .002; Figure 5B).

**FIGURE 6:**
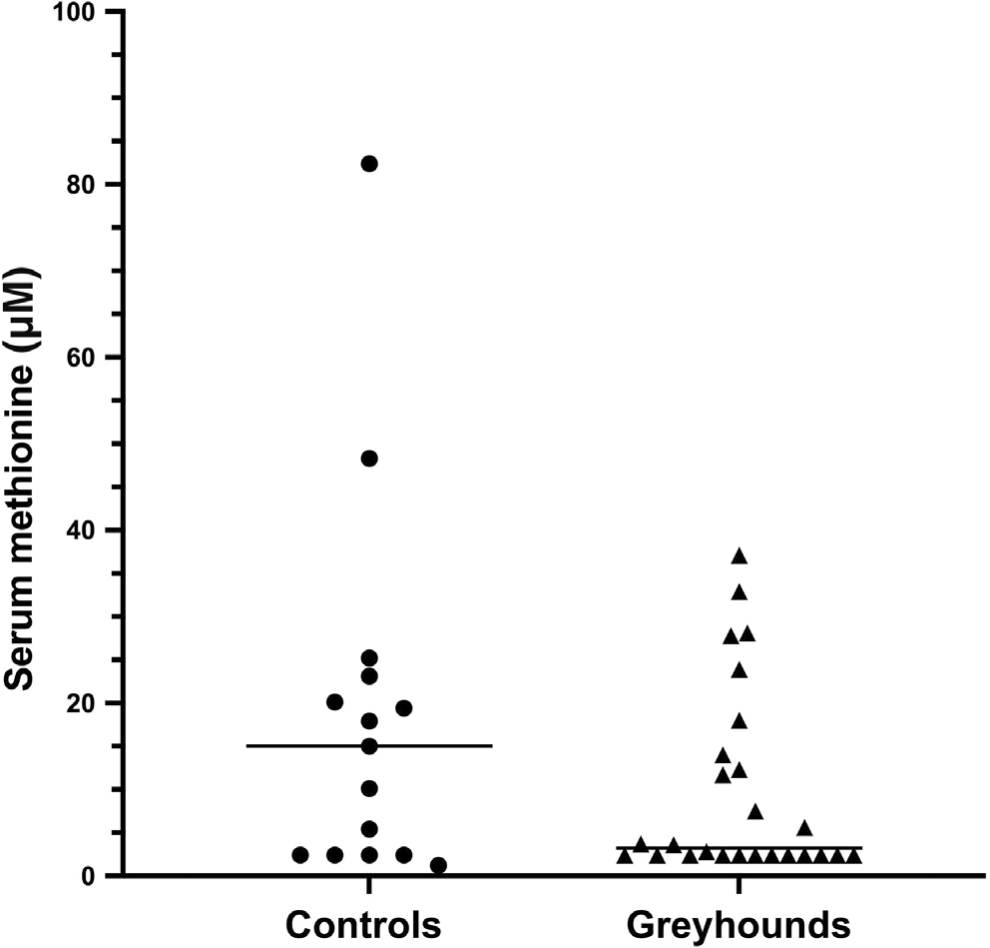
Serum methionine concentrations in pet Greyhound dogs and non‐sighthound controls. Median concentrations were more than 4‐fold lower in Greyhounds but were not significantly different (*P* = .3), possibly because of unexpected heterogeneity in the control group.

Serum methionine concentrations were observed to be more than 4‐fold lower in Greyhounds (median, 3.2 μmol/L; range, 2.4‐37.10 μmol/L) compared to controls (median, 15.0 μmol/L; range, 1.2‐82.4 μmol/L), but this difference was not significant (*P* = .30), possibly because of heterogeneity in the control group (Figure 6). Many Greyhounds had concentrations below the limit of detection of the methionine assay (2.5 μmol/L), but low serum methionine concentrations were not correlated with high serum HCy concentrations in this population (*r* = −0.06, *P* = .73).

### 3.4 Plasma 8‐isoprostanes

**FIGURE 7:**
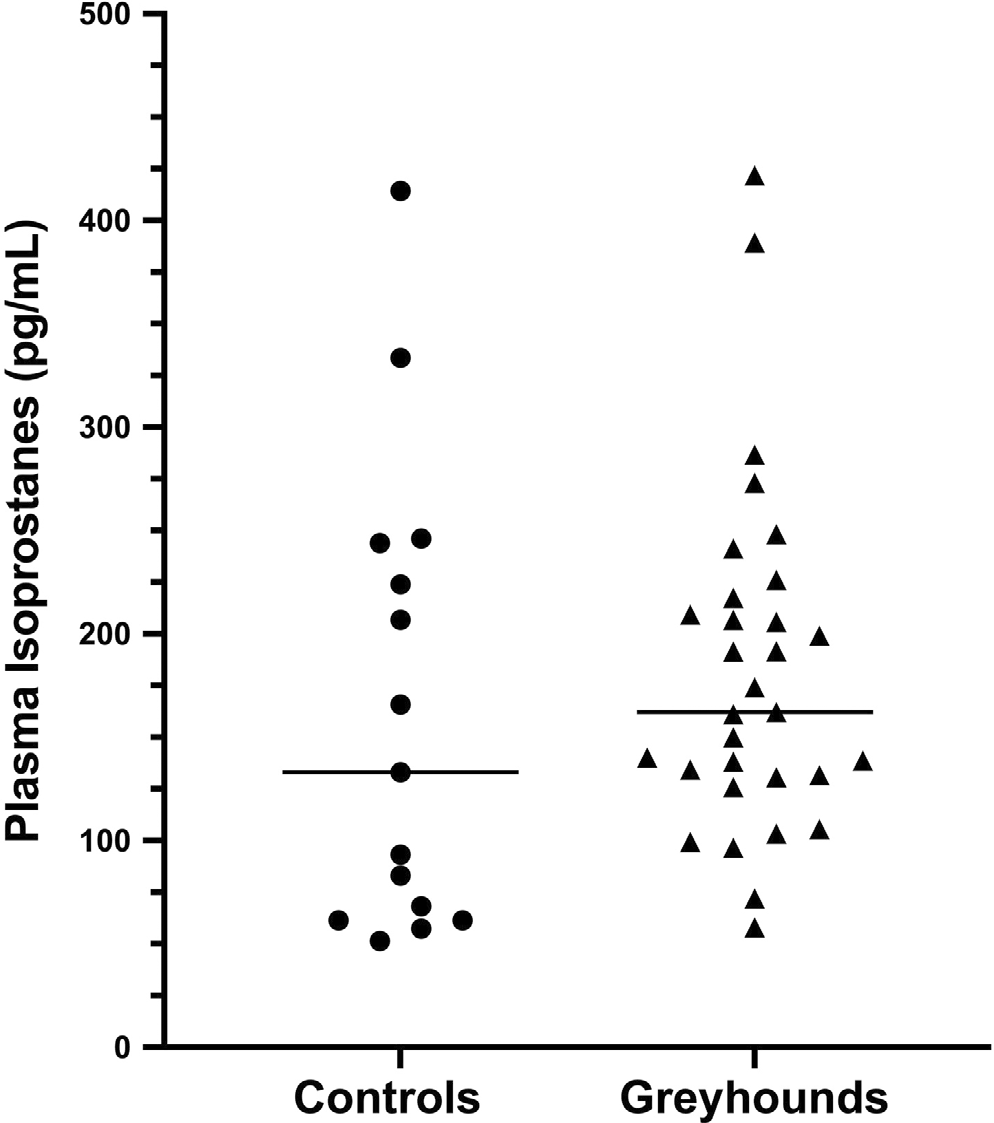
Total plasma 8‐isoprostane concentrations, as a measure of oxidative stress, in pet Greyhound dogs and non‐sighthound controls, which were not significantly different between groups (*P* = .31).

Median plasma isoprostane concentrations were not significantly higher in Greyhound dogs (162 pg/mL; range, 58‐422 pg/mL) compared to controls (133 pg/mL; range, 51‐414 pg/mL; *P* = .31; Figure 7). Furthermore, plasma 8‐isoprostane concentrations did not correlate with serum HCy concentrations across all dogs (*r* = 0.03, *P* = .86) or across Greyhounds (*r* = −0.14, *P* = .46).

## 4 DISCUSSION

Our study confirmed the presence of HHC in Greyhounds, which previously was reported in a heterogenous group of Greyhound dogs, 16 of which were reported to be clinically healthy.^15^ We found that serum HCy concentrations were significantly higher in screened healthy Greyhounds (median, 25 μmol/L) compared to non‐sighthound control dogs (13.9 μmol/L). In the previous study of 16 clinically healthy Greyhounds,^15^ median HCy concentrations were even higher (approximately 65 μmol/L), with 4‐fold variability. In both studies, all healthy Greyhounds had supranormal HCy concentrations (>22 μmol/L). However, although the dogs in our study had complete medical histories, physical examinations, and normal baseline laboratory test results to be considered for inclusion, we still found more than 6‐fold variability in HCy concentrations among healthy Greyhounds, suggesting modifying factors other than breed. One of these factors is likely to be increased age, as demonstrated by the positive correlation between age and HCy concentrations across Greyhound dogs.

When we assessed serum cobalamin concentrations, the Greyhounds in our study had significantly lower serum concentrations compared to matched non‐sighthound dogs. Nearly 40% of the Greyhounds in our study (12 of 31) had cobalamin concentrations that generally are considered low enough to warrant supplementation based on current recommendations from the Gastrointestinal Laboratory at Texas A&M University. Serum cobalamin concentrations in Greyhounds also were significantly and inversely correlated with HCy concentrations in our study. This relationship was not found in the previous study,^15^ possibly because of the smaller population of healthy Greyhounds. Hyperhomocysteinemia has been reported in Shar Peis and Giant Schnauzers with breed‐related hypocobalaminemic disorders.^14^, ^25^ These findings indicate that primary cobalamin deficiency can drive HHC in some dogs. In our Greyhounds, however, only 1 dog had a serum cobalamin concentration below the lower limit of the reference interval, suggesting that low serum cobalamin concentrations are unlikely to be the primary driver of HHC in Greyhounds. However, cobalamin deficiency can occur at the cellular level even with normal serum cobalamin concentrations,^26^ and cobalamin supplementation can improve HCC even in patients without overt hypocobalaminemia.^27^

Serum folate concentrations were below the lower limit of the reference interval (7.7 μg/L) in approximately 55% (16 of 29) of the healthy Greyhounds in our study. Although not significantly different from controls, serum folate concentrations were inversely correlated with serum HCy concentrations. This relationship also was noted in the previous group of Greyhounds.^15^ Folate is generated by conversion of HCy to methionine (Figure 1), and thus low serum folate concentrations support an impairment in this pathway.

We also measured serum methionine concentrations, which were 3‐fold lower in Greyhounds compared to non‐sighthound dogs. Although this difference was not significant because of heterogeneity within breed groups, this finding supports a Greyhound defect in the conversion of HCy to methionine, either by methionine synthase or its recycling enzyme, methionine synthase reductase. Cobalamin is an important cofactor for this pathway, and thus modest deficiencies in cobalamin could further exacerbate a genetic defect in either methionine synthase or methionine synthase reductase.^28^

The observation of subnormal serum cobalamin and folate concentrations in some healthy Greyhounds without gastrointestinal signs is important to consider when using these analytes as markers for small intestinal disease. Low serum cobalamin concentrations in Greyhounds could be a result of unrecognized intestinal malabsorption but also could be caused by depletion from an ineffective cycling of methionine synthase.

No significant differences were found between groups in plasma cysteine or glutathione concentrations, which does not support impaired conversion of HCy to cysteine as a mechanism of HHC in Greyhounds. Also, no difference was found in plasma 8‐isoprostane concentrations between breed groups, which does not support the hypothesis that HHC leads to systemic oxidative stress in otherwise healthy Greyhounds. This finding differs from previous studies in people where HHC has been associated with increases in plasma 8‐isoprostane concentrations.^6^, ^7^, ^8^, ^9^ We used a commercially available competitive enzyme immunoassay to measure plasma isoprostanes, which has successfully detected increases in plasma oxidative stress in dogs.^29^, ^30^ However, this assay has not been fully validated in canine plasma, and subsequent studies in Greyhounds should incorporate a broader array of oxidative stress markers.

Although our study has strengths in its prospective screening and use of non‐sighthound dogs as controls, it had some limitations. First, measurement of methylmalonic acid was not included in our study, and thus we were unable to assess cobalamin deficiency on a cellular level.^14^ Second, supportive documents for the folate assay (Immulite 2000 Folic Acid, Siemens Healthcare Diagnostics) suggest that the assay does not differentiate between tetrahydrofolate (THF) and 5‐methyl THF, making it difficult to fully assess the methylenetetrahydrofolate reductase (MTHFR) pathway (Figure 1).

In addition, although each dog in the study ate a consistent diet, the diets varied among individual dogs. The population size was small, and we may have lacked power to detect significant differences in serum methionine concentrations between breed groups. Finally, although HHC is associated with a prothrombotic state in people,^5^ prospective measurement of coagulation parameters was outside the scope of our study.

Overall, our findings suggest a primary defect in conversion of HCy to methionine in otherwise healthy Greyhounds. We did not find evidence of oxidative stress associated with HHC in this breed using plasma 8‐isoprostane concentrations, but additional measures of oxidative stress should be evaluated. Because HHC can lead to prothrombotic states in people, an assessment of coagulation status is needed in relation to HCy concentrations in Greyhounds. In addition, resequencing of the methionine synthase (*MTR*) and methionine synthase reductase (*MTRR*) genes should be performed in Greyhounds to determine whether coding variants are related to inter‐ and intrabreed variability in serum HCy concentrations. Supplementation with B vitamins can improve HCC even in human patients with genetic defects in the conversion of HCy to methionine,^11^, ^27^ and supplementation would be an important treatment option to be studied if HHC were shown to be associated with adverse outcomes in Greyhound dogs.

## CONFLICT OF INTEREST DECLARATION

Joerg M. Steiner is the Director of the Gastroenterology Laboratory at Texas A&M University, which provides fee‐for‐service assays for some of the analyses included in this study. Lauren A. Trepanier serves as Associate Editor for the Journal of Veterinary Internal Medicine. She was not involved in review of this manuscript. No other authors declare a conflict of interest.

## OFF‐LABEL ANTIMICROBIAL DECLARATION

Authors declare no off‐label use of antimicrobials.

## INSTITUTIONAL ANIMAL CARE AND USE COMMITTEE (IACUC) OR OTHER APPROVAL DECLARATION

Approved by the University of Wisconsin‐Madison IACUC, protocol V006303‐A01.

## HUMAN ETHICS APPROVAL DECLARATION

Authors declare human ethics approval was not needed for this study.
